# Kisspeptin upregulates β-cell serotonin production during pregnancy

**DOI:** 10.1530/JOE-23-0218

**Published:** 2023-12-18

**Authors:** Thomas G Hill, Lorna I F Smith, Inmaculada Ruz-Maldonado, Peter M Jones, James E Bowe

**Affiliations:** 1 Oxford Centre for Diabetes, Endocrinology and Metabolism, Radcliffe Department of Medicine, Churchill Hospital, University of Oxford, Oxford, UK; 2 Diabetes Research Group, School of Cardiovascular and Metabolic Medicine and Sciences, King’s College London, London, UK; 3 Department of Internal Medicine (Endocrinology), Yale University, New Haven, Connecticut, USA

**Keywords:** islets, serotonin, pregnancy, kisspeptin, insulin

## Abstract

During pregnancy the maternal pancreatic islets of Langerhans undergo adaptive changes to compensate for gestational insulin resistance. The lactogenic hormones are well established to play a key role in regulating the islet adaptation to pregnancy, and one of the mechanisms through which they act is through upregulating β-cell serotonin production. During pregnancy islet serotonin levels are significantly elevated, where it is released from the β-cells to drive the adaptive response through paracrine and autocrine effects. We have previously shown that placental kisspeptin (KP) also plays a role in promoting the elevated insulin secretion and β-cell proliferation observed during pregnancy, although the precise mechanisms involved are unclear. In the present study we investigated the effects of KP on expression of pro-proliferative genes and serotonin biosynthesis within rodent islets. Whilst KP had limited effect on pro-proliferative gene expression at the time points tested, KP did significantly stimulate expression of the serotonin biosynthesis enzyme Tph-1. Furthermore, the islets of pregnant β-cell-specific GPR54 knockdown mice were found to contain significantly fewer serotonin-positive β-cells when compared to pregnant controls. Our previous studies suggested that reduced placental kisspeptin production, with consequent impaired kisspeptin-dependent β-cell compensation, may be a factor in the development of GDM in humans. These current data suggest that, similar to the lactogenic hormones, KP may also contribute to serotonin biosynthesis and subsequent islet signalling during pregnancy. Furthermore, upregulation of serotonin biosynthesis may represent a common mechanism through which multiple signals might influence the islet adaptation to pregnancy.

## Introduction

Pregnancy is characterised by a shift in maternal metabolism with a progressive increase in maternal insulin resistance to prioritise nutrient flow to the growing fetus (Battaglia & Meschia 1978, Hay 1991, Baumann *et al.* 2002, Freemark 2006, Lain & Catalano 2007, Baeyens *et al.* 2016). This rise in insulin resistance is balanced by a compensatory increase in the insulin secretory capacity of the β-cells within the islets of Langerhans (Van Assche *et al.* 1978, Parsons *et al.* 1992, Sorenson & Brelje 1997, Butler *et al.* 2010), which is achieved through several mechanisms, including increased β-cell mass, increased insulin synthesis and heightened sensitivity to glucose stimulation (Bowe *et al.* 2019) of insulin secretion. Failure of the maternal β-cells to adapt to this heightened metabolic demand may result in glucose intolerance and gestational diabetes (GDM), a condition that currently affects ~7% of all pregnancies worldwide and is associated with a variety of adverse outcomes to both the mother and offspring (Alberti & Zimmet 1998, Metzger *et al.* 2010, Rani & Begum 2016, American Diabetes Association 2017, Zheng *et al.* 2018).

Our understanding of the signals regulating the β-cell adaptive changes during gestation are not fully understood. It is well established that the lactogenic hormones, prolactin (Prl) and placental lactogen (PL), play an important role in driving β-cell proliferation and increasing glucose-stimulated insulin secretion (GSIS) during pregnancy (Sorenson *et al.* 1987, Parsons *et al.* 1992, Moldrup *et al.* 1993, Weinhaus *et al.* 1996, 2007, Bole-Feysot *et al.* 1998, Vasavada *et al.* 2000, 2006, Friedrichsen *et al.* 2001, 2003, Brelje *et al.* 2002, Huang *et al.* 2009, Sorenson & Brelje 2009, Arumugam *et al.* 2011, 2014, Banerjee *et al.* 2016). These changes are achieved through several intracellular mechanism, including an upregulation of mitogenic cell cyclin regulators (e.g. Ccnd1), anti-apoptotic/pro-survival targets such as Birc5 (Friedrichsen *et al.* 2001, 2003, Karnik *et al.* 2007, Rieck *et al.* 2009, Rieck & Kaestner 2010, Arumugam *et al.* 2011, Xu *et al.* 2015), and the two rate-limiting serotonin biosynthesis enzymes, tryptophan hydroxylase 1 (Tph-1) and 2 (Tph-2) (Rieck *et al.* 2009, Rieck & Kaestner 2010, Schraenen *et al.* 2010). Serotonin released from the islets during pregnancy plays an important role in driving proliferative and pro-survival adaptations through autocrine/paracrine signalling (Kim *et al.* 2010, Schraenen *et al.* 2010, Ohara-Imaizumi *et al.* 2013, Almaca *et al.* 2016, Moon *et al.* 2020).

In addition to the established role of the lactogenic hormones, we have previously demonstrated a role for kisspeptin signalling in the islet adaptation to pregnancy. The kisspeptins (kisspeptin 54, kisspeptin 14, kisspeptin 13 and kisspeptin 10) are a family of peptides that act as the endogenous ligands for the G_α__q_-protein-coupled receptor GPR54 (also commonly known as KISS1R) (Kotani *et al.* 2001, Muir *et al.* 2001, Ohtaki *et al.* 2001). Kisspeptin is best known for its permissive effects in regulating the onset of puberty and reproductive function through actions in the hypothalamus (De Roux *et al.* 2003, Dhillo *et al.* 2005, 2007, Topaloglu *et al.* 2012, Kirilov *et al.* 2013). However, kisspeptin and GPR54 are also highly expressed in several peripheral tissues (Muir *et al.* 2001, Ohtaki *et al.* 2001, Seminara *et al.* 2003) including the pancreatic islets (Hauge-Evans *et al.* 2006, Song *et al.* 2014). We and others have reported stimulatory effects for exogenous kisspeptin on insulin secretion from rodent, porcine, and human islets *in vitro*, as well as within rodents, non-human primates and humans *in vivo* (Hauge-Evans *et al.* 2006, Bowe *et al.* 2009, 2012, Wahab *et al.* 2011, Schwetz *et al.* 2014, Andreozzi *et al.* 2017, Izzi-Engbeaya *et al.* 2018). Kisspeptin is also abundantly expressed within the human and rodent placenta (placental syncytiotrophoblasts in humans and large trophoblastic cells in rodents) and whilst physiological circulating kisspeptin levels are normally very low (<2 pM), they are dramatically elevated (~1000–7000-fold) during human gestation, suggesting that kisspeptin/GPR54 may also be involved in regulating glucose homeostasis during pregnancy (Horikoshi *et al.* 2003, Bilban *et al.* 2004, Dhillo *et al.* 2006, Cetkovic *et al.* 2012, Mark *et al.* 2013, Herreboudt *et al.* 2015, Drynda *et al.* 2018). We have previously demonstrated that endogenous kisspeptin/GPR54 plays an important role in the adaptation of maternal β-cells to pregnancy by amplifying β-cell GSIS and β-cell replication (Bowe *et al.* 2019), but the intracellular mechanisms underlying these effects are unclear.

Here we report that kisspeptin/GPR54 signalling in rodents is also linked to a downstream upregulation of islet *Tph-1* and *Ccnd1* mRNA expression, as well as an increase in β-cell serotonin production. These findings suggest that some of the kisspeptin/GPR54-induced adaptive changes in β-cell proliferation during pregnancy may be indirectly mediated by an increase in islet autocrine/paracrine serotonin signalling, and further support the importance of placental-derived kisspeptin in the islet adaptation to pregnancy.

## Materials and methods

### Animals

Female ICR mice (Envigo, Bicester, UK) at 8 weeks of age were used for *in vivo* pharmacological studies. Mice were either maintained as non-pregnant controls or mated with male ICRs and checked daily for the presence of vaginal plugs to establish pregnancy. Non-pregnant mice were implanted with subcutaneous osmotic minipumps loaded with 1 mM of kisspeptin 10 (minipumps: Alzet Corp., Cupertino, CA, kisspeptin 10: Alta Biosciences, Redditch, UK) under isoflurane anaesthesia as previously described (Bowe *et al.* 2019). In pregnant mice minipumps loaded with 1 mM of kisspeptin antagonist, kisspeptin 234, were implanted at day 8 of pregnancy. Osmotic minipumps released 0.25 nmol/h at an infusion rate of 0.4 nmol/h.

The β-cell GPR54^−/−^ mouse model was generated on a C57Bl/6 background using Cre-Lox recombination. Briefly, GPR54-LoxP mice (obtained from Prof. Tena-Sempere, University of Cordoba, Spain) were cross-bred with MIP-Cre/ERT mice (B6.Cg-Tg(Ins1-cre/ERT)1Lphi/J, Jackson Labs). Tamoxifen was administered to induce GPR54 knockdown in the relevant mice at 8 weeks of age through daily intraperitoneal (i.p.) injection (100 µL of 20 mg/mL tamoxifen in peanut oil, Sigma-Aldrich) for 4 consecutive days. Mice were then left for 6 weeks to allow tamoxifen washout. Further details and validation of the model has been described previously (Bowe *et al.* 2019).

All animals were housed under controlled conditions (14 h light–10 h darkness; lights on at 07:00 h; temperature at 22 ± 2°C) and provided with food and water *ad libitum*. All animal experiments were conducted using ARRIVE guidelines in accordance with the United Kingdom Animal (Scientific Procedures) Act 1986. The studies were approved by the relevant local ethical review committee (King’s College London, Guy’s Campus Animal Welfare and Ethical Review Body) and conducted under the authority of the Project Licence PBCFBE464.

### Islet isolation and treatment

Islets of Langerhans were isolated from ICR mice by collagenase digestion of the exocrine pancreas and incubated at 37°C in RPMI (supplemented with 10% fetal calf serum, 2 mM glutamine and 100 U/mL penicillin/0.1 mg/mL streptomycin) for at least 24 h before use.

Groups of 200 islets were handpicked and incubated for 48 h in RPMI-1640 (supplemented with 10% (v/v) FBS and 1% (v/v) penicillin/streptomycin containing either sub-stimulatory (2 mM) or stimulatory (20 mM) d-glucose (Sigma-Aldrich) in the presence or absence of 1 μM mouse kisspeptin 10 (Alta Biosciences, Redditch, Worcestershire, UK). Following incubation, treated islets were pelleted by centrifugation (200 ***g***, 2 min) incubated in RLT lysis buffer (supplemented with 5% (v/v) β-mercaptoethanol), and snap frozen in liquid N_2_. Samples were stored at −80°C until required for RNA extraction.

### RNA extraction

Total RNA from isolated islets were extracted from homogenates using a QIAshredder column and RNeasy mini kit (Qiagen), and followed according to the manufacturer’s instructions. Any potential contaminating DNA was removed using a DNAse 1 treatment kit (Qiagen). The resulting RNA concentration and purity was assessed utilising a NanoDrop 1000 spectrophotometer (ThermoFisher Scientific). Likewise, the integrity of RNA samples was checked and visualised via agarose (2% w/v) gel electrophoresis and ethidium bromide staining (0.5 μg/mL). Samples were stored at −80°C until required for cDNA synthesis.

### Reverse transcription

Approximately 500 ng of total RNA from each sample were reverse transcribed into cDNA using a Multiscribe™ Reverse Transcription kit (Applied Biosystems), containing a recombinant Moloney murine leukaemia virus reverse transcriptase and random dNTP primers, and followed according to the manufacturer’s instructions using a four-step thermal cycler program: pre-incubation, 25°C (10 min); synthesis, 37°C (120 min); heat inactivation, 85°C (5 min); cooling 4°C (10 min). The resulting cDNA samples were stored at −20°C until required for qPCR.

### Quantitative real-time polymerase chain reaction

The mRNA expression of select genes were quantified using a LightCycler^®^ 480 real-time qPCR system in a 96-well plate format as previously described (Amisten *et al.* 2015). PCR mixtures consisting of Qiagen^®^ Quanitfast SYBR Green mastermix (5 μL). RNase/DNase-free water (2 μL) and designed gene-specific cDNA forward and reverse primers (2 μL) were prepared and mixed with 2 μL of the target cDNA of interest. Wells consisting of primers and SYBR Green master mix alone were utilised as negative controls. The mRNA expression of each gene of interest was normalised with reference to the mRNA expression of the internal housekeeping genes, murine *Gapdh* and *β-actin,* using validated Qiagen QuantiTect^®^ primers. Relative quantification was assessed utilising the ΔΔCT method. Melting curve analysis for each amplified sample was conducted following each PCR for positive target identification.

### Fluorescent immunohistochemistry

Pancreata taken from ICR mice implanted with osmotic minipumps and β-cell GPR54^−/−^ mice were fixed in 4% paraformaldehyde (818715, Merck Millipore) and embedded in paraffin wax. For immunohistochemical staining sections (5 µm) were rehydrated followed by antigen retrieval with citrate buffer (10 mM citric acid (anhydrous), 0.05% Tween 20, pH 6.0) in a pressure cooker for 2 min. Non-specific binding was blocked using a blocking buffer (0.25% BSA, 0.25% Triton X-100 in PBS) prior to incubation with either polyclonal guinea pig anti-insulin (1:200) along with either monoclonal rat anti-serotonin (sc-73024, Santa Cruz Biotechnology; 1:200) or monoclonal mouse anti-GPR54 (ab221859, Abcam; 1:100) primary antibodies diluted in blocking buffer overnight at 4°C. Sections incubated with blocking buffer alone were used as negative controls.

Alexa-594 conjugated donkey anti-guinea pig (1:100, Jackson Immuno Research) was used to visualise insulin staining. Alexa-488 conjugated donkey anti-mouse (1:100, Jackson Immuno Research) was used to visualise GPR54 whilst either Alexa 488 or Cy5 conjugated donkey anti-rat (1:100, Jackson Immuno Research) were used to visualise serotonin immunostaining. All sections were mounted with a fluorescent mounting medium (for sections undergoing CellProfiler analysis, DAPI Fluoromount; Cambridge Bioscience Ltd., was used) and visualised using a Nikon Eclipse TE2000-U microscope and Nikon DS-Qi1MC camera.

Sections were analysed from across the pancreas for each animal and all islets on each section were analysed. For quantitative serotonin analysis, all islets from each section were identified and cropped using ImageJ image analysis software, before input into CellProfiler software (Broad Institute, Inc., Cambridge, MA, USA). Here, the software used DAPI and insulin staining to segment the islet into individual cells (labelled CytoObjects, see Supplementary data, see section on supplementary materials given at the end of this article). These objects were then filtered by insulin intensity to remove insulin-negative, non-β-cells from the analysis (see Supplementary data). Cells identified by the software as β-cells (labelled InsObjects) were then counted and their serotonin integrated intensity measured. The integrated intensity of serotonin staining in all β-cells was calculated and given that the staining intensity is not normally distributed an upper quartile threshold was set for identifying serotonin-positive cells. It was also confirmed that there was no significant difference in the number of islets or β-cells analysed per treatment group, nor the mean total insulin intensity measured per section (see Supplementary data).

### Statistical analysis

All numerical data are expressed as the mean ± s.e.m. An unpaired two-tailed Student’s *t*-test was used for comparisons between two groups. A one-way analysis of variance followed by Tukey’s *post* *hoc* honestly significantly different multiple comparisons test was used for comparisons between three or more groups. Statistical analysis was assessed using Prism Graphpad statistical software. Differences with *P* < 0.05 were considered significant.

## Results

### Exposure of mouse islets to kisspeptin *in vitro* increases the mRNA expression level of key cell cycle regulator/proliferative genes

**Figure 1:**
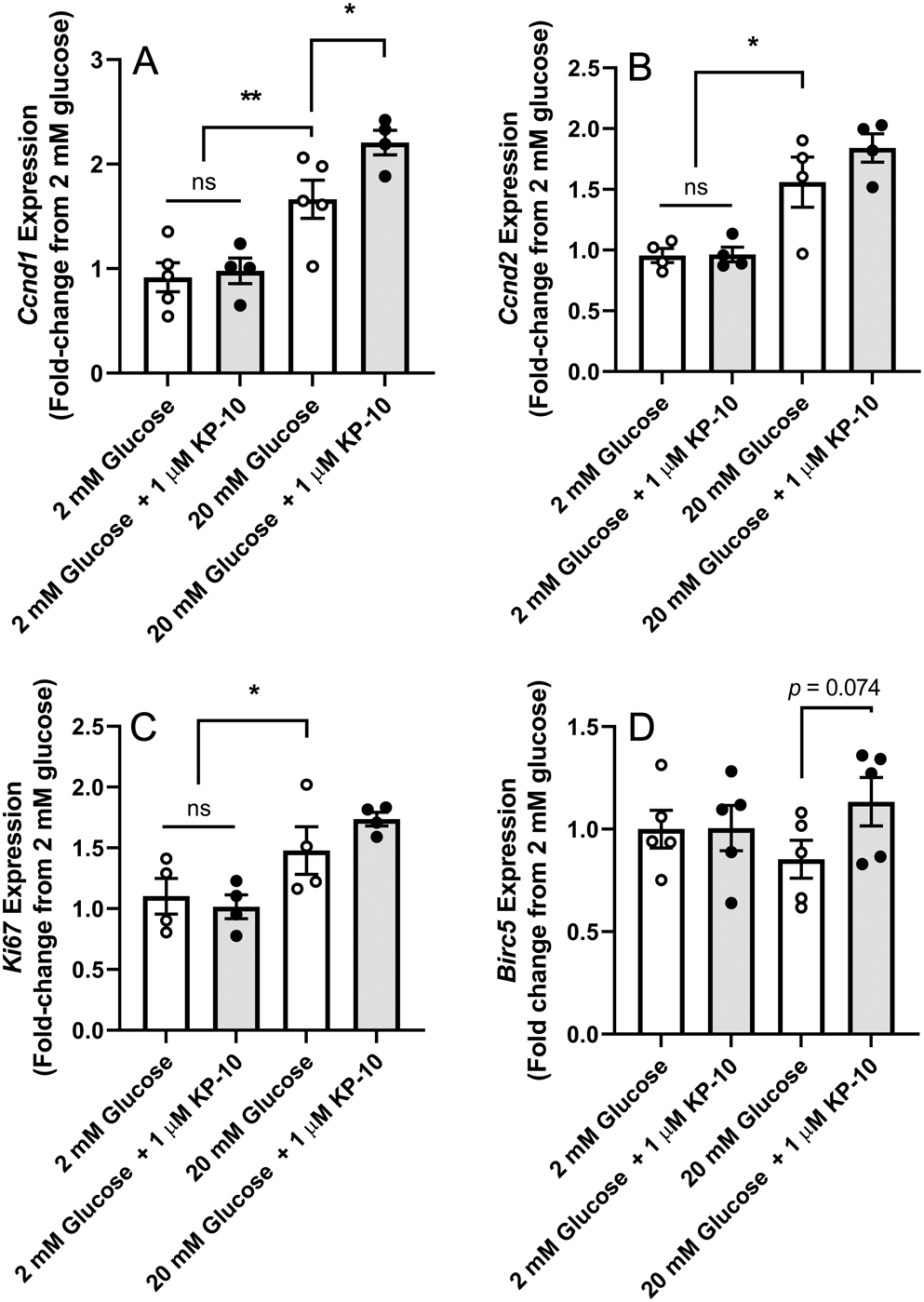
Effect of exogenous kisspeptin on the mRNA expression of select cell cycle genes within murine islets *in vitro*. Quantitative PCR was performed on cDNA extracted from ICR islets cultured *in vitro* with either sub-stimulatory (2 mM) or stimulatory (20 mM) glucose in the presence (black) or absence (white) of exogenous kisspeptin 10 (1 μM) for 48 h. Data represent the mean fold-change in mRNA expression ± s.e.m. as compared with the 2 mM glucose control group. *n* = 5–8 pooled replicates for each treatment, with each replicate containing 200 islets pooled from 5-6 age-matched mice. *P < 0.05 and **P < 0.01, using a one-way ANOVA followed by a Tukey’s *post* *hoc* test.

We have previously shown that a loss of endogenous kisspeptin signalling during pregnancy reduces β-cell proliferation *in vivo* (Bowe *et al.* 2019), but the intracellular mechanisms involved are unclear. We chronically treated isolated non-pregnant (ICR) islets with either low (2 mM) or high (20 mM) glucose in the presence or absence of 1 µM kisspeptin 10 for 48 h and assessed the transcript expression of key cell cycle regulating/proliferative (*Ccnd1*, *Ccnd2, Ki67*) and pro-survival (*Birc5*) genes *in vitro* using qPCR (Figs. 1A, B, C and D). The exposure of isolated non-pregnant islets to stimulatory (20 mM) glucose for 48 h triggered significant increases in *Ccnd1*, *Ccnd2* and *Ki67* mRNA expression, when compared to non-pregnant islets cultured in 2 mM glucose (Figs. 1A, B and C). The addition of exogenous kisspeptin 10 (1 µM) induced a significant 32% amplification in *Ccnd1* when cultured at 20 mM glucose, compared to islets cultured at 20 mM glucose alone (Fig. 1A). There was also a slight 17-18% increase in *Ccnd2* and *Ki67* mRNA expression in response to kisspeptin, though these were not significant (Figs. 1B and C respectively). Neither glucose nor exogenous kisspeptin 10 had any significant effect on *Birc5* expression (Fig. 1D).

### Exogenous kisspeptin increases the mRNA expression of the serotonin biosynthesis enzyme, Tph-1 within the β-cells of non-pregnant mouse islets

**Figure 2:**
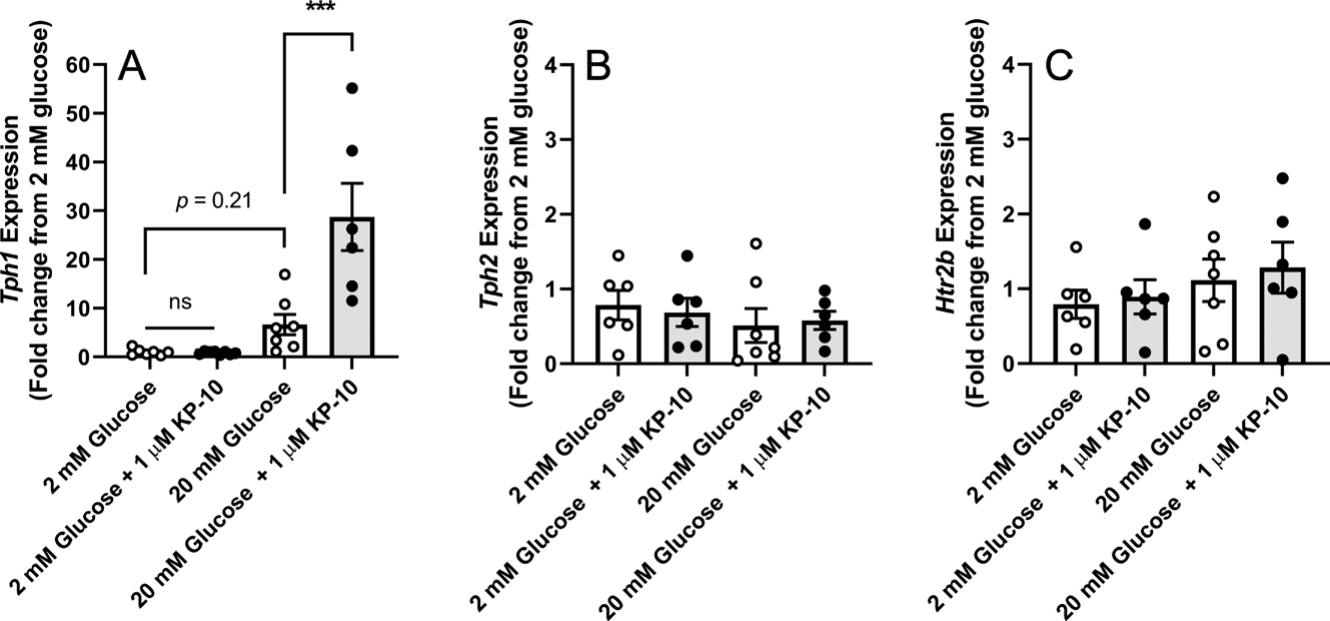
Effect of exogenous kisspeptin on the mRNA expression of serotonin biosynthetic enzymes within murine islets *in vitro*. Quantitative PCR was performed on ICR islet cDNA cultured *in vitro* with 2 mM or 20 mM glucose in the presence (black) or absence (white) of exogenous kisspeptin 10 (1 μM) for 48 h. Data represent the mean fold-change in mRNA expression ± s.e.m. compared to 2 mM glucose control islets. *n* = 5–8 pooled replicates for each treatment, with each replicate containing 200 islets pooled from five to six age-matched mice. *P < 0.05 and **P < 0.01, using a one-way ANOVA followed by a Tukey’s *post* *hoc* test.

Due to the involvement of serotonin signalling in mediating the effects of lactogenic hormones on β-cell replication during pregnancy we investigated whether islet kisspeptin exposure altered the mRNA expression of the serotonin biosynthesis enzymes tryptophan hydroxylase-1 (*Tph-1*) and -2 (*Tph-2*). Chronic treatment of non-pregnant islets with exogenous kisspeptin 10 significantly upregulated the mRNA expression of *Tph-1* by 4.32-fold when cultured in 20 mM glucose, when compared to islets cultured in 20 mM glucose alone (Fig. 2A). In contrast to *Tph-1*, no change in *Tph-*2 expression (Fig. 2B), or expression of the Gα_q_-coupled serotonin receptor, 5-hydroxytryptamine-2b (*Htr2b*, Fig. 2C) was found within non-pregnant islets treated with kisspeptin 10 when cultured at either glucose concentration.

### Effect of kisspeptin signalling on β-cell serotonin synthesis during pregnancy

To determine whether chronic kisspeptin exposure also causes an increase in β-cell serotonin production *in vivo*, we initially performed fluorescent immunohistochemistry utilising a validated anti-serotonin antibody on pancreatic sections taken from non-pregnant virgin or GD18 pregnant female ICR mice. Non pregnant mice were chronically implanted with subcutaneous osmotic minipumps containing kisspeptin 10 to mimic elevated kisspeptin levels during pregnancy or saline for 10 days. Conversely, pregnant mice were chronically administered with the kisspeptin antagonist, kisspeptin 234, to block the endogenous kisspeptin during pregnancy or saline via subcutaneous osmotic minipump from day 8 to day 18 of pregnancy.

**Figure 3:**
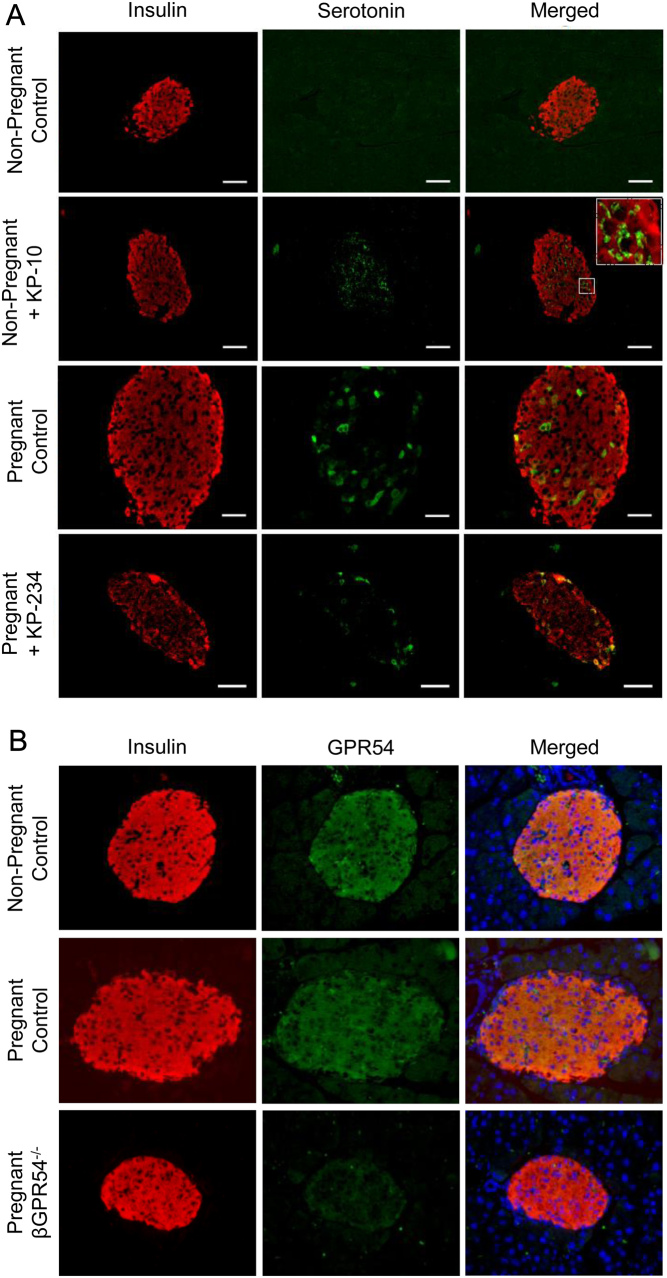
Effect of chronic *in vivo* exposure of non-pregnant islets to exogenous kisspeptin and pregnant islets to GPR54 antagonist, kisspeptin 234, on islet serotonin. (A) Non-pregnant and pregnant ICR female mice were chronically administered either with exogenous kisspeptin 10 (0.25 nM/h) or with GPR54 antagonist, kisspeptin 234, respectively, *in vivo* for 10 days via subcutaneous osmotic mini-pump. Osmotic mini-pumps containing saline alone were used as controls. GD18 pancreas sections were immunoprobed for both insulin (red) and serotonin (green). (B) Sections from non-pregnant and pregnant female control Cre+/TMX- as well as non-pregnant β-cell GPR54^−/−^ mice were also immunoprobed for either insulin (red) with GPR54 (green). Scale bar = 50 µm.

Islets from non-pregnant female mice chronically treated with exogenous kisspeptin 10 showed an increase in β-cell serotonin staining when compared to untreated non-pregnant control islets (Fig. 3A), suggesting that chronic exposure of kisspeptin 10 *in vivo* causes an increase in islet β-cell serotonin production. As expected, the pancreatic islets from pregnant saline-administered controls displayed strong cellular serotonin staining (green) that was localised to a subpopulation of islet β-cells (red) when compared to islets from saline-administered non-pregnant controls. The islets of pregnant mice chronically treated with the GPR54 antagonist, kisspeptin 234 still retained cellular serotonin immunofluorescence localised to the islet β-cells when compared to islets from saline-administered pregnant controls, but the number of serotonin-positive cells was lower when compared to pregnant controls. Unlike the pattern of serotonin production, GPR54 is expressed almost ubiquitously in both non-pregnant and pregnant β-cells (Fig. 3B).

**Figure 4:**
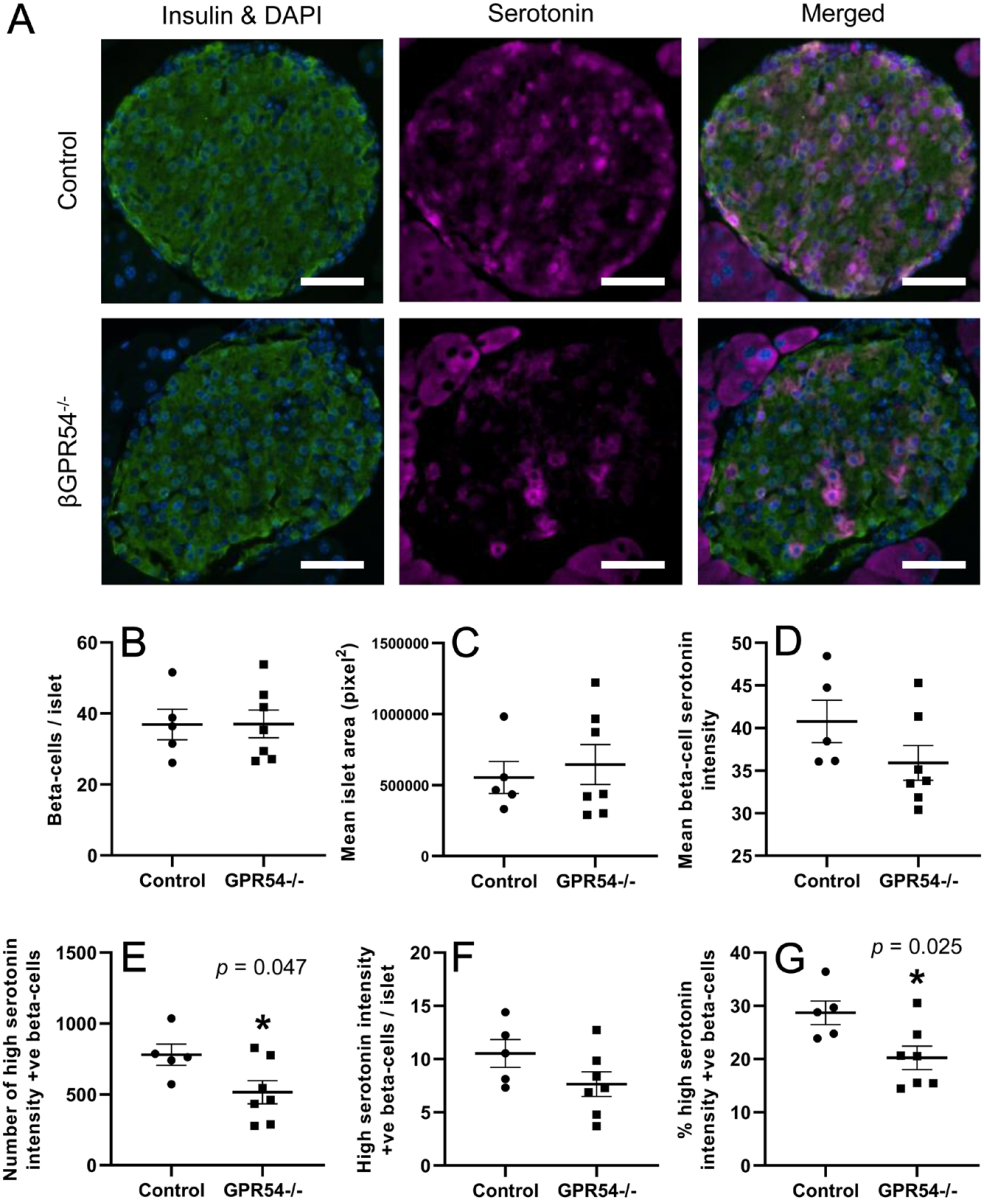
Effect of endogenous kisspeptin on islet serotonin during pregnancy. Representative illustrative images of immunostaining in pregnant (A) control Cre+/TMX- and β-cell GPR54^−/−^ islets showing insulin (green) and DAPI (blue), serotonin (pink) and merged images. (B, C and D) β-cell GPR54^−/−^ mice had no significant differences in the number of β-cells per islet, mean islet area or the overall serotonin staining intensity compared to pregnant control mice. Scale bar = 50 µm. When β-cells expressing serotonin immunostaining intensity above an upper quartile threshold were identified there were significantly fewer total serotonin-positive β-cells identified per animal (E) and a significantly lower percentage of β-cells identified as serotonin positive (G) in β-cell GPR54^−/−^ islets compared to controls, although the difference in number of serotonin-positive β-cells per islet (F) was not significantly different. Mean ± s.e.m., *n* = 5–7, *P < 0.05.

Based on these initial observations we analysed islets from β-cell specific GPR54^−/−^ mice for more detailed investigation of whether endogenous kisspeptin may play a role in regulating islet serotonin synthesis during pregnancy. We have previously reported that islets from βGPR54^−/−^ mice have significantly reduced islet GPR54 mRNA expression and that is reflected in the reduced GPR54 immunostaining observed in βGPR54^−/−^ islets (Fig. 3B). Pancreas samples were taken at day 18 of pregnancy from βGPR54^−/−^ mice and controls. Cre+ve mice without tamoxifen induction of GPR54 knockdown were used as controls as described in our previous study. No significant differences in β-cell numbers per islet, nor islet area were seen between control and βGPR54^−/−^ mice (Figs. 4B and C), consistent with our previous studies (Bowe *et al.* 2019). Mean serotonin intensity per β-cell indicated a decrease in serotonin synthesis in βGPR54^−/−^ mice compared to controls though this was not significant (control: 40.77 ± 2.49, βGPR54^−/−^: 35.90 ± 2.05, *P* = 0.16). Upon further investigation we identified that mean serotonin intensity was not normally distributed amongst β-cells, with the vast majority of β-cells displaying very low serotonin production. Therefore, for each section, β-cells which expressed over the upper quartile of mean serotonin synthesis in an experimental run were counted as ‘high serotonin intensity positive β-cells’. Upon completion of this analysis, we found that the total number of β-cells with high serotonin intensity was significantly lower in pancreas sections from βGPR54^−/−^ mice compared to controls (Fig. 4E, control: 778.9 ± 74.47, βGPR54^−/−^: 515.2 ± 82.23, *P* = 0.047). Additionally, the overall percentage of β-cells with high serotonin intensity was significantly lower in βGPR54^−/−^ mice (Fig. 4G, control: 28.70 ± 2.23%, βGPR54^−/−^: 20.25 ± 2.20%, *P* = 0.025).

## Discussion

Under most physiological conditions circulating kisspeptin levels are very low; however, during pregnancy kisspeptin is abundantly expressed and released from the placenta into the maternal circulation. In human pregnancy circulating kisspeptin can reach levels that are several 1000-fold higher than in non-pregnant conditions (Dhillo *et al.* 2006, Panidis *et al.* 2006). Kisspeptin levels in rodents are less clear due to the lack of commercially reliable assays; however, we and others have reported Kiss-1 mRNA to also be expressed and upregulated within the rodent placenta (Mark *et al.* 2013, Herreboudt *et al.* 2015, Drynda *et al.* 2018). Whilst the physiological purpose of this placental kisspeptin is not fully understood, we have previously demonstrated that endogenous kisspeptin plays an important role in placenta–islet communication to mediate the islet adaptation to pregnancy.

Pregnancy is characterised by a progressive increase in peripheral insulin resistance from mid-to-late pregnancy (GDs 10-18 in mice), which is counteracted by an improvement in β-cell function and increase in β-cell mass (Spellacy & Goetz 1963, Van Assche *et al.* 1978, Brelje & Sorenson 1991, Sorenson & Brelje 1997, Butler *et al.* 2010, Baeyens *et al.* 2016). Loss of β-cell kisspeptin signalling in pregnant mice results in both reduced insulin secretion in response to glucose challenge and in reduced β-cell proliferation resulting in significantly impaired glucose tolerance compared to control pregnant mice. In humans, women diagnosed with GDM have significantly lower plasma kisspeptin when compared to normoglycaemic healthy controls, identifying kisspeptin as a potential biomarker for the clinical diagnosis of GDM (Bowe *et al.* 2019). The intracellular mechanisms through which endogenous kisspeptin might potentiate glucose-induced insulin secretion have been studied previously. We have shown that kisspeptin increases intracellular Ca^2+^ levels in β-cells and that the increased insulin secretion is dependent on PLC signalling. These mechanisms are consistent with other studies showing activation of the Gα_q/11_ G-protein by GPR54, signalling via a classical Gα_q_-coupled IP3-PLC-Ca^2+^ signalling pathway in several cell culture systems (Kotani *et al.* 2001, Stafford *et al.* 2002). However, the mechanisms involved in kisspeptin action on β-cell proliferation are less clear.

During adulthood, the islet β-cells are normally mitotically ‘quiescent’ with a turnover rate of approximately 0.2% and 2% per day in humans and rodents, respectively (Bonner-Weir *et al.* 1989, Bonner-Weir 2000). However, during pregnancy, rodent and human β-cells undergo an increase in β-cell mass highlighting their adaptive plasticity in response to metabolic stress (Van Assche *et al.* 1978, Butler *et al.* 2010, Rieck & Kaestner 2010, Baeyens *et al.* 2016). In rodents, this rise in β-cell mass is mainly attributed to an increase in β-cell proliferation and hypertrophy which occurs primarily after placentation but is reversible post partum, suggesting placental signals to mediate an important role in this adaptable change (Parsons *et al.* 1992, Weinhaus *et al.* 1996, Sorenson & Brelje 1997). It is well-established that in rodents that the lactogenic hormones acting on the β-cell Prlr and downstream activation of transcription factor Stat5, trigger an increase in β-cell proliferation *in vitro* and *in vivo* via the upregulation of the cell cyclin genes, such as Ccnd1, and anti-apoptotic signals, such as Birc-5 and Bcl-xL (Brelje *et al.* 1993, Brelje *et al.* 2002, Friedrichsen *et al.* 2003, Bordin *et al.* 2004, Rieck *et al.* 2009, Rieck & Kaestner 2010, Arumugam *et al.* 2011, Xu *et al.* 2015). Our recent *in vivo* studies showed β-cells from pregnant βGPR54^−/−^ mice contain reduced BrdU incorporation (an S-phase marker for cell proliferation) when compared to pregnant littermate controls at gestational day 18 (Bowe *et al.* 2019). Here we find that exposure of non-pregnant mouse islets to kisspeptin *in vitro* also causes a significant upregulation in *Ccnd1* transcript expression, suggesting that kisspeptin may activate similar intracellular proliferative mechanisms to those activated in response to the lactogenic hormones.

Consistent with previous reports in rodent islets (Arumugam *et al.* 2011) and INS-1 cells (Asfari *et al.* 1995), the mRNA expression of *Ccnd1*, *Ccnd2* and *Ki67* were prominently upregulated within islets cultured in stimulatory (20 mM) glucose when compared to islets cultured in sub-stimulatory glucose (2 mM) alone, confirming an influential role for glucose itself in driving the G1/S cell cycle progression within β-cells. The effects of kisspeptin on *Ccnd1* expression is only apparent in the presence of stimulatory glucose, with no impact on *Ccnd1* expression in islets cultured in 2 mM glucose. This observation is consistent with the actions of prolactin on isolated islets, where elevated glucose concentrations significantly increased the effects of prolactin on mRNA expression of proliferative markers (Arumugam *et al.* 2011). However, unlike prolactin, kisspeptin did not significantly affect the expression levels of other genes associated with the pregnancy-related increase in β-cell proliferation such as *Ccnd2*, *Ki67* and *Birc5*.

It is possible that changes in the expression of these genes were not detected due to the 48 h treatment period being insufficient. However, prolactin treatment does induce changes in proliferation-related markers in this time-frame. Whilst important, it is unclear how much endogenous β-cell kisspeptin/GPR54 signalling contributes to regulating islet adaptation during pregnancy relative to other signals. As previously reported, a loss of endogenous kisspeptin signalling has a more modest effect on β-cell proliferation than the equivalent effect seen in response to PRLR knockdown. Furthermore, pregnant βGPR54^−/−^ mice and pregnant mice chronically infused with a GPR54 antagonist still exhibit significantly elevated β-cell proliferation when compared to non-pregnant controls. It is possible that the effects of kisspeptin on pro-proliferative pathways may be more subtle and difficult to detect, or may not involve activation of all the factors associated with PRLR. Regardless, the significant upregulation of *Ccnd1* expression supports the previous conclusions that kisspeptin plays a role in stimulating β-cell proliferation during pregnancy.

Interestingly, in addition to a significant upregulation of *Ccnd1* expression, kisspeptin also induced a significant increase in the expression of the serotonin biosynthesis enzyme *Tph1* (Fig. 2A). Whilst β-cell serotonin is normally low, studies over the last decade have shown that during pregnancy the lactogenic hormones induce an upregulation in islet Tph1/2 expression and a consequent increase in β-cell serotonin production (Rieck *et al.* 2009, Kim *et al.* 2010, Schraenen *et al.* 2010, Ohara-Imaizumi *et al.* 2013). This increase in β-cell serotonin synthesis is not consistent across all β-cells, rather a select sub-population of β-cells become serotonin positive to different degrees during pregnancy (Schraenen *et al.* 2010). This increased β-cell serotonin is released and acts in a local autocrine/paracrine role via the Htr2b receptor, upregulating cell cyclin expression and contributing to the compensatory β-cell changes that occur during gestation (Kim *et al.* 2010). Local serotonin release is also believed to contribute to increased insulin secretion through Htr3, a serotonin-gated ion channel (Ohara-Imaizumi *et al.* 2013). Similar to previous reports on lactogenic signalling, we show that exposure of non-pregnant islets to exogenous kisspeptin *in vitro* for 48 h elicits a significant upregulation in islet *Tph-1* mRNA expression (Fig. 2A). Initial *in vivo* investigations found that islets of non-pregnant female mice chronically administered kisspeptin showed an increase in islet serotonin synthesis within a subpopulation of β-cells, whilst islets of pregnant mice chronically administered with KP-234, a kisspeptin antagonist, exhibited serotonin immunostaining in fewer β-cells (Fig. 3). The observation that serotonin synthesis occurs in a subpopulation of β-cells is consistent with previous studies, although it is worth noting that GPR54 is widely expressed throughout the β-cells in both non-pregnant and pregnant mice (Fig. 3B). It is well-established that the β-cells are heterogenous, but the specific properties of this subpopulation of serotonin producing β-cells are unclear. Given the suggested role of serotonin in driving pregnant β-cell proliferation it would be interesting to determine whether these cells are more highly proliferative themselves or drive the proliferation of nearby cells. However, for the purposes of the present study the upregulation of serotonin synthesis in only specific β-cells in response to kisspeptin does not appear to be due to selective expression of GPR54 in this subpopulation of β-cells.

These observations were confirmed in more detailed analysis of pregnant βGPR54^−/−^ mouse islets. We have previously shown a reduction in β-cell proliferation in pregnant βGPR54^−/−^ mice compared to controls, though no significant effect on overall islet area (Bowe *et al.* 2019). The present study confirms the lack of significant effect on islet area but importantly identifies that βGPR54^−/−^ mice have a reduced number of serotonin-positive β-cells during pregnancy. Specifically, a reduced percentage of βGPR54^−/−^ β-cells contain serotonin staining intensity above a set threshold (upper quartile for overall mean β-cell staining intensity). It is worth noting that when serotonin producing cells are identified using a much higher intensity threshold then in the data presented here (twice the value of the upper quartile) there is no significant difference in the percentage of βGPR54^−/−^ β-cells containing serotonin (data not shown). This suggests that kisspeptin does not impact the small number of β-cells that very highly synthesise serotonin but instead significantly influences the larger number of β-cells that exhibit moderate serotonin activity.

These observations are the first evidence linking kisspeptin signalling to serotonin synthesis in the β-cell, although several previous studies have demonstrated an association between kisspeptin and serotonin in other tissues and systems. In the zebrafish kisspeptin treatment has been shown to increase serotonin levels in whole brain samples (Sivalingam *et al.* 2020), and habenular kisspeptin neurones are specifically linked to an increase in serotonin activity in the raphe nucleus associated with fear responses (Ogawa *et al.* 2012, Nathan *et al.* 2015*a*,*b*). Unlike our results in the β-cell implying a direct effect of kisspeptin to upregulate serotonin biosynthesis, the effects of kisspeptin on raphe nucleus serotonin in the zebrafish appear to be mediated at least in part via a glutamergic and/or GABAergic pathway (Nathan *et al.* 2015*b*, Ogawa & Parhar 2018). However, direct effects of habenular kisspeptin neurones on the zebrafish raphe nucleus serotonin system have not been ruled out. The effects of kisspeptin on serotonin activity in rodents are less studied and poorly understood. Kisspeptin 10 has been shown to inhibit serotonin biosynthesis in the Hypo-E22 hypothalamic cell line (Orlando *et al.* 2018), whilst in the rat kisspeptin treatment does not appear to have any detectable effect on serotonin release or turnover in the median eminence (Ribeiro *et al.* 2015). However, several studies have identified an association between kisspeptin and serotonin in models of depressive behaviours, which suggest that kisspeptin may upregulate serotonin activity in some brain regions. Kisspeptin administration has been shown to have anti-depressant effects in both rats and humans (Tanaka *et al.* 2013, Comninos *et al.* 2017), as well as a role in passive avoidance learning in mice (Telegdy & Adamik 2013). Both the anti-depressant effects of kisspeptin in the rat and the effects on avoidance learning in the mouse can be blocked through administration of 5-HT2 serotonergic receptor antagonists, strongly suggesting that these actions of kisspeptin are mediated through stimulation of serotonin systems (Tanaka *et al.* 2013, Telegdy & Adamik 2013).

Despite the relatively limited research literature, there is clear precedent for kisspeptin exerting physiological effects though modulating the serotonin system. The findings of the present study suggest that the kisspeptin-driven effects on upregulated *Ccnd1* expression *in vitro* and reported β-cell proliferation during pregnancy may be mediated indirectly by an increase in intra-islet serotonin signalling. Serotonin is expressed by the islet β-cells and β-cell serotonin/Htr2b signalling has recently been reported to regulate perinatal β-cell development and the crucial maintenance of β-cell mass/glucose tolerance during adulthood by stimulating β-cell proliferation (Moon *et al.* 2020), it is likely that kisspeptin-induced β-cell serotonin also contributes to proper perinatal β-cell proliferation and mass expansion. It is well established that stimulation of islet serotonin synthesis by the lactogenic hormones plays an important role in mediating the pregnancy-associated increases in β-cell mass, whilst disruption to local serotonin production or signalling during pregnancy results in impaired islet adaptation and subsequent glucose intolerance. Our results suggest that reduced serotonin synthesis due to lack of kisspeptin signalling has similar consequences to those observed in response to a lack of prolactin signalling. Thus, these data indicate that β-cell serotonin may represent a common mediator through which multiple endocrine signals may act to achieve the overall effect of increasing β-cell mass during pregnancy.

It is also worth noting that serotonin may also potentially play some role in mediating the effects of kisspeptin on insulin secretion. We have previously shown that kisspeptin potentiates glucose-induced insulin secretion and that this effect is mediated through increased intracellular Ca^2+^ (Bowe *et al.* 2009). The βGPR54^−/−^ mice have normal islet insulin content and glucose-induced insulin secretion, but kisspeptin potentiation of insulin secretion is lost (Bowe *et al.* 2019). Whilst these data are consistent with direct effects through GPR54, a G_q_-coupled receptor, serotonin also increases insulin secretory responses, but not capacity, during pregnancy through Htr3 (Ohara-Imaizumi *et al.* 2013) and may potentially play an indirect role in influencing insulin release.

## Conclusions

In conclusion, we have demonstrated that treatment of isolated islets with exogenous kisspeptin causes an increase in cell cyclin *Ccnd1* transcript expression. We have also shown that exposure of islets to exogenous kisspeptin increases islet *Tph-1* transcript *in vitro* and serotonin synthesis *in vivo*. More significantly, disruption of endogenous kisspeptin signalling during pregnancy results in reduced islet serotonin biosynthesis. Given the role that islet β-cell proliferation and serotonin signalling plays during rodent gestation, these studies support our previous findings highlighting the importance of kisspeptin as an important novel agent in the maintenance of normal β-cell proliferation and function during pregnancy.

## Supplementary Materials

Supplementary data: Representative illustrative images of the stages of CellProfiler analysis for the measurement of islet serotonin content. Initially DAPI staining is analysed and all cell nuclei are identified as NucObjects (A, B and C). Insulin staining is subsequently analysed and in combination with previously identified nuclei all β-cells are identified as CytoObjects (D, E and F). Finally serotonin staining is analysed and β-cells containing serotonin immunostaining above a given threshold are identified (G, H and I). There were no significant differences in the total number of islets analysed, the total number of β-cells analysed, or the insulin intensity between β-cell GPR54−/− islets compared to controls.

## Declaration of interest

The authors declare that there is no conflict of interest that could be perceived as prejudicing the impartiality of the research reported.

## Funding

This study was supported by grant funding from both the Wellcome Trusthttp://dx.doi.org/10.13039/100010269 (grant number 108064/Z/15/Z) and the Biotechnology and Biological Sciences Research Councilhttp://dx.doi.org/10.13039/501100000268 (grant number BB/N00616X/1). For the purpose of open access, the authors have applied a Creative Commons Attribution (CC BY) licence to any Author Accepted Manuscript version arising.
