# Supplementary figures and images for: Kisspeptin upregulates β-cell serotonin production during pregnancy
Source: J Endocrinol. 2023 Dec 18;260(2):e230218. doi: 10.1530/JOE-23-0218 (PMC10762540; doi:10.1530/JOE-23-0218)

# Supplementary data

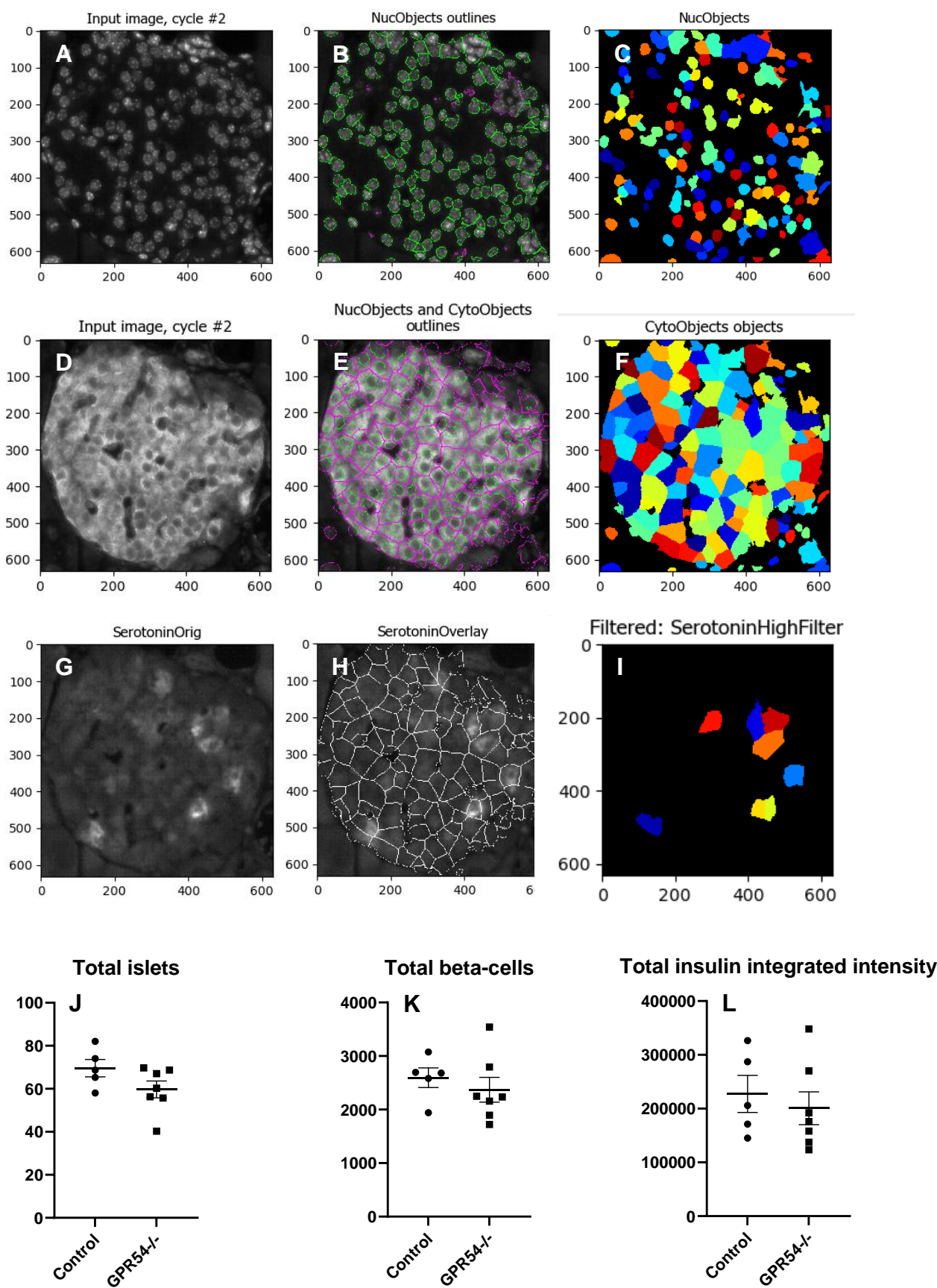

Supplement: Supplementary data: Representative illustrative images of the stages of CellProfiler analysis for the measurement of islet serotonin content. Initially DAPI staining is analysed and all cell nuclei are identified as NucObjects (A, B and C). Insulin staining is subsequently analysed and in combinatio [file supplementary_data.pdf]
